# Neisseria meningitidis: The Unforeseen Agent of Acute Neonatal Conjunctivitis

**DOI:** 10.7759/cureus.65681

**Published:** 2024-07-29

**Authors:** Catarina Albuquerque, Mariana E Dias, Marta Pelicano, Denise Banganho, Rita B Morais

**Affiliations:** 1 Pediatrics, Hospital de São Francisco Xavier, Unidade Local de Saúde de Lisboa Ocidental, Lisbon, PRT

**Keywords:** infection microbiology, anti-infective agents, newborn and child health, neisseria meningitidis, neonatal conjunctivitis

## Abstract

Acute conjunctivitis is a common disease in the neonatal period. Although often underestimated, *Neisseria meningitidis* is an uncommon but potentially severe cause of acute neonatal conjunctivitis. We describe a case of a 14-day-old healthy female newborn who presented with fever, runny nose, cough, and bilateral purulent ocular discharge. A nasopharyngeal swab tested positive for SARS-CoV-2, and the infant was discharged after becoming afebrile 24 hours later. Four days later, ocular exudate culture revealed the presence of *N. meningitidis* and *Staphylococcus aureus*. Blood and cerebrospinal fluid tests were unremarkable. The infant was treated with intravenous cefotaxime and topical azithromycin, with no signs of invasive disease or reported complications. This case highlights noninvasive neonatal acute conjunctivitis caused by a coinfection of *N. meningitidis* and *S. aureus*, with a favorable outcome. The ocular exudate culture was crucial in identifying the causative bacteria, which might otherwise have gone undetected and improperly treated. Clinicians should consider *N. meningitidis* as a potential agent in neonatal acute conjunctivitis.

## Introduction

Acute conjunctivitis is a common condition in the neonatal period, occurring in approximately 1-12% of newborns within the first four weeks of life. The primary causes of acute neonatal conjunctivitis are chemical, bacterial, or viral agents. These infectious agents can be transmitted during vaginal delivery through passage in the birth canal or in cesarean delivery via ascending infection of the uterus. Key risk factors for neonatal conjunctival infection due to vertical transmission include premature rupture of membranes, maternal infection, and inadequately monitored pregnancies [1].

While often underestimated,* Neisseria meningitidis* is an unusual but serious cause of acute conjunctivitis, with only a few cases reported [2,3]. It is estimated to account for 1-2% of all cases of acute neonatal conjunctivitis. The presumed route of transmission for primary infections is intrapartum, but airborne or contact transmission can also lead to secondary infections [2-5]. Asymptomatic carriers of *N. meningitidis*, particularly among children and young adults, have been identified through positive vaginal or nasopharyngeal swabs [3-5]. Conjunctival infections caused by *N. meningitidis* can progress to an invasive disease in 10-29% of cases and may lead to significant ocular complications, including corneal ulcers, keratitis, subconjunctival hemorrhage, and iritis [2,3].

Systemic therapy, combined with topical antibiotics, is recommended for treatment. Current guidelines consider ceftriaxone to be the empiric gold standard. For noninvasive infections, systemic antibiotics should be administered for at least five days. It is also crucial to identify close contacts and initiate chemoprophylaxis as needed [3].

## Case presentation

A 14-day-old female newborn was admitted to the emergency department with a fever. The parents were young adults, and the pregnancy had been unremarkable, with negative maternal serologies for hepatitis B and C, human immunodeficiency virus, syphilis, and *Toxoplasma gondii*, and normal fetal ultrasounds. During the third trimester, the mother had shown polymicrobial growth in a urine culture but did not receive treatment, and the vaginal swab for Group B *Streptococcus* had been negative. Vaginal delivery occurred at 41 weeks in a tertiary hospital, with membranes rupturing 12 hours before birth and clear amniotic fluid. There was no record of maternal fever. The baby was born weighing 2,730 grams (small for gestational age), with an Apgar score of 9 at one minute and 10 at five minutes. She did not receive ophthalmic prophylaxis, in accordance with the hospital’s protocol. The mother and child were discharged after two days.

Upon admission, the newborn was febrile (axillary temperature of 38 °C), and presented with a runny nose, cough, and reduced food intake. There were no urinary or gastrointestinal symptoms. The mother reported similar symptoms. Clinical examination revealed a weight gain of 35 grams in the previous four days and bilateral purulent eye discharge. The remainder of the examination was unremarkable. Laboratory findings showed: hemoglobin 15.9 g/dL, leukocytes 10.2 × 10^9^/L, neutrophils 3.06 × 10^9^/L, lymphocytes 3.37 × 10^9^/L, platelets 491 × 10^9^/L, C-reactive protein 2.3 mg/L, and procalcitonin 0.16 ng/mL. Renal function and the electrolyte panel were age-appropriate, as detailed in Table 1.

**Table 1:** Blood work results on first admission

Blood work	First admission
Hemoglobin (g/dL)	15.9
Leukocytes (× 10^9^/L)	10.2
Neutrophils (× 10^9^/L)	3.06
Lymphocytes (× 10^9^/L)	3.37
Platelets (× 10^9^/L)	491
C-reactive protein (mg/L)/procalcitonin (ng/mL)	2.3/0.16
Glucose (mg/dL)	108
Urea/creatinine (mg/dL)	20/0.2
Sodium (mmol/L)/potassium (mmol/L)	137/5.0

Due to her age, she was hospitalized for surveillance. Ocular exudate was collected, and she was started on topical ocular azithromycin. The nasopharyngeal swab tested positive for SARS-CoV-2. Blood and urine cultures were negative. The microbiological test results are described in Table 2.

**Table 2:** Microbiological test results on first admission PCR: polymerase chain reaction; RSV: respiratory syncytial virus; RT-PCR: reverse transcription polymerase chain reaction

Microbiological tests	First admission
Blood culture	Negative
Urine culture	Negative
SARS-CoV-2 (PCR, nasopharyngeal swab)	Positive
Influenza A/B (RT-PCR, nasopharyngeal swab)	Negative
RSV (RT-PCR, nasopharyngeal swab)	Negative

She remained apyretic, with adequate food intake and no signs of systemic disease, so she was discharged after 24 hours. Four days later, the ocular exudate culture tested positive for *N. meningitidis *and *Staphylococcus aureus*, while the PCR test was negative for *Neisseria gonorrhoeae *and *Chlamydia trachomatis*. Given the risk of invasive disease, the infant was readmitted to the hospital for intravenous antibiotic treatment. On readmission, she had shown appropriate weight gain since discharge (60 grams per day), minimal exudate in the left eye, no palpebral edema or conjunctival hyperemia, and no other signs of disease. Blood tests, blood cultures, and a lumbar puncture were performed. Blood work revealed no abnormalities (Table 3): hemoglobin 14.7 g/dL, leukocytes 10.9 × 10^9^/L, neutrophils 1.14 × 10^9^/L, lymphocytes 8.39 × 10^9^/L, platelets 617 × 10^9^/L, glucose 80 mg/dL, C-reactive protein <1.0 mg/L, and procalcitonin 0.07 ng/mL.

**Table 3:** Blood work results on second admission

Blood work	Second admission
Hemoglobin (g/dL)	14.7
Leukocytes (× 10^9^/L)	10.9
Neutrophils (× 10^9^/L)	1.14
Lymphocytes (× 10^9^/L)	8.39
Platelets (× 10^9^/L)	617
C-reactive protein (mg/L)/procalcitonin (ng/mL)	<1.0/0.07
Glucose (mg/dL)	80
Urea/creatinine (mg/dL)	18/<0.17
Sodium (mmol/L)/potassium (mmol/L)	137/4.5

The cerebrospinal fluid was hemorrhagic, with protein levels of 74 mg/dL, glucose of 42 mg/dL, and 70 mononuclear cells/µL. She was started on cefotaxime 200 mg/kg/day. Blood and cerebrospinal fluid cultures were negative. The microbiological test results are summarized in Table 4.

**Table 4:** Microbiological test results on second admission CMV: cytomegalovirus; HHV: human herpesvirus 6; HSV: herpes simplex virus; PCR: polymerase chain reaction; VZV: varicella zoster virus

Microbiological tests	Second admission
Blood culture	Negative
Cerebrospinal fluid culture	Negative
Meningitis/encephalitis pathogen panel (multiplex PCR)	Negative for HSV-1 and HSV-2; HHV-6; CMV; VZV; enterovirus; human parechovirus; *Neisseria meningitidis*; *Streptococcus pneumoniae*; *Streptococcus agalactiae*; *Listeria monocytogenes*; *Escherichia coli *K1; *Haemophilus influenzae*; and *Cryptococcus neoformans/gattii*

The susceptibility test (Table 5) indicated that the* N. meningitidis *isolate was susceptible to cefotaxime, meropenem, and penicillin, while the* S. aureus *isolate was susceptible to oxacillin and gentamicin (methicillin-susceptible *S. aureus*, MSSA).

**Table 5:** Susceptibility test results for ocular exudate culture

Ocular exudate culture	*Neisseria meningitidis*	*Staphylococcus aureus*
Cefotaxime	Susceptible	
Erythromycin		Susceptible
Fusidic acid		Susceptible
Gentamicin		Susceptible
Meropenem	Susceptible	
Oxacillin		Susceptible
Benzylpenicillin	Susceptible	
Tetracycline		Susceptible

She completed a seven-day course of intravenous cefotaxime. A favorable clinical response was observed, with complete resolution of the exudate and no detected complications. The three households, all asymptomatic, received prophylactic antibiotics: adults were given a single dose of 500 mg ciprofloxacin, while the 16-year-olds received rifampicin at 10 mg/kg every 12 hours for two days. The case was reported to the local public health services.

## Discussion

Acute conjunctivitis is common among neonates. During this period, sexually transmitted agents must be considered due to their potential severity and association with long-term complications. *N. gonorrhoeae *and *C. trachomatis *are the primary pathogens responsible. However, other agents also require systemic treatment, including *N. meningitidis*, which, although uncommon, should be considered. Neonatal prophylaxis remains controversial: while it may have significantly reduced the incidence of *N. gonorrhoeae infections *in developed countries, severe gonococcal acute conjunctivitis is rare. Furthermore, antibiotic prophylaxis has not yet been proven to prevent serious outcomes [6]. The antibiotic regimen varies: silver nitrate, which caused chemical conjunctivitis, is no longer available, making erythromycin ophthalmic ointment the most feasible option, although it shows limited effectiveness against *Chlamydia *species [7]. Consequently, ocular antibiotic prophylaxis has been questioned, and universal prophylaxis is not mandatory in some European countries [8].

Conjunctival infection caused by *N. meningitidis *typically presents with abundant unilateral purulent ocular discharge, which resembles the presentation of *N. gonorrhoeae* [2]. In our case, the scant bilateral exudate was atypical, resembling infections caused by other common bacterial agents such as *S. aureus *or *Haemophilus influenzae*. The purulent discharge was found to be positive for two agents, marking what we believe to be the first reported case of coinfection with *N. meningitidis*. This underscores the critical role of ocular exudate culture in neonatal conjunctivitis in identifying causative bacteria that might otherwise remain undetected.

The risk of invasive disease with *N. meningitidis *is significant, as systemic symptoms can develop up to 96 hours after the onset of ocular exudate [2]. To rule out invasive disease, we deemed it necessary to perform a lumbar puncture, despite the absence of clinical signs of systemic involvement. The highest risk of invasive disease occurs within the first four days after the onset of conjunctivitis, highlighting the importance of early Gram staining and initiating empirical systemic treatment. Although oral therapy for *N. meningitidis *conjunctivitis is mentioned in the literature, it carries a higher risk of invasive disease, making intravenous therapy the preferred option [3]. Ceftriaxone is generally the antibiotic of choice; however, due to the infant’s age, we used cefotaxime. The child showed a positive therapeutic response after two days of treatment, suggesting that cefotaxime is a viable alternative to ceftriaxone for treating *N. meningitidis *acute neonatal conjunctivitis.

It was our practice to collect ocular exudates from all neonates with acute conjunctivitis in the emergency room. We performed culture and PCR tests for *N. gonorrhoeae *and *C. trachomatis *and used azithromycin for empirical topical treatment, which showed a good in vivo response in most cases.

The source of infection in our case was not investigated. Given the two- to five-day incubation period for *N. meningitidis* [9] and the presentation on the 14th day of life, we presumed the infection may have been linked to late secondary transmission via air droplets or direct contact with an asymptomatic carrier. In such cases, it could be indicated to collect vaginal and nasopharyngeal swabs from both parents [5,10]. The age of cohabitants might have been a risk factor for infection. To prevent further transmission, chemoprophylaxis was administered to close contacts. Fortunately, no complications or additional transmissions were reported during or after this episode.

## Conclusions

We describe a case of noninvasive neonatal acute conjunctivitis caused by a coinfection with *N. meningitidis*, an uncommon pathogen, and MSSA, a more frequent microorganism. The presentation was atypical, and the microbiological identification was crucial for initiating early, appropriate systemic therapy during the initial days of the illness, when the risk of invasiveness is highest. The incidence of *N. meningitidis *conjunctivitis may be underestimated due to the absence of conjunctival exudate cultures in all affected neonates. This diagnosis can be challenging, potentially delaying treatment and increasing the risk of invasive disease or long-term complications. Clinicians should consider *N. meningitidis *as a potential cause of neonatal acute conjunctivitis.

## References

[1] Sawyer T, Gleason CA (2023). Avery's Diseases of the Newborn. Avery's Diseases of.

[2] Orden B, Martínez R, Millán R, Belloso M, Pérez N (2003). Primary meningococcal conjunctivitis. Clin Microbiol Infect.

[3] Parikh SR, Campbell H, Mandal S, Ramsay ME, Ladhani SN (2019). Primary meningococcal conjunctivitis: summary of evidence for the clinical and public health management of cases and close contacts. J Infect.

[4] Filippakis D, Gkentzi D, Dimitriou G, Karatza A (2022). Neonatal meningococcal disease: an update. J Matern Fetal Neonatal Med.

[5] Fiorito SM, Galarza PG, Sparo M, Pagano EI, Oviedo CI (2001). An unusual transmission of Neisseria meningitidis: neonatal conjunctivitis acquired at delivery from the mother's endocervical infection. Sex Transm Dis.

[6] Kapoor VS, Evans JR, Vedula SS (2020). Interventions for preventing ophthalmia neonatorum. Cochrane Database Syst Rev.

[7] Smith-Norowitz TA, Ukaegbu C, Kohlhoff S, Hammerschlag MR (2021). Neonatal prophylaxis with antibiotic containing ointments does not reduce incidence of chlamydial conjunctivitis in newborns. BMC Infect Dis.

[8] (2024). Cuidados Gerais ao Recém-Nascido Saudável. https://www.spneonatologia.pt/wp-content/uploads/2023/08/Cuidados-gerais-ao-RN-Saud%C3%A1vel.pdf..

[9] (2018). Red Book (2018): Report of the Committee on Infectious Diseases. Diseases AAPCoI. Red Book ( 2018): Report of the Committee on Infectious Diseases: American Academy of Pediatrics.

[10] Chacon-Cruz E, Alvelais-Palacios JA, Rodriguez-Valencia JA, Lopatynsky-Reyes EZ, Volker-Soberanes ML, Rivas-Landeros RM (2017). Meningococcal neonatal purulent conjunctivitis/sepsis and asymptomatic carriage of N. meningitidis in mother's vagina and both parents' nasopharynx. Case Rep Infect Dis.

